# 260 Triple-lumen Hemodialysis Catheters Reduce the Prevalence of Concurrent Central Venous Catheters in Patients Admitted to the ICU

**DOI:** 10.1017/ash.2026.10626

**Published:** 2026-06-23

**Authors:** Bushra Shaik Ismail, Ameer S/O Anaith Ali, Molly How, Lee Rachel, Moi Lin Ling

**Affiliations:** 1 Singapore General Hospital

## Abstract

**Introduction:** Hand hygiene compliance in healthcare settings is significantly influenced by the acceptability and skin tolerability of hand sanitisers. Conventional alcohol-based hand rubs have been associated with increasing incidences of skin dryness, irritation, and contact dermatitis with repeated application, leading to reduced compliance and limited alternatives for staff with skin sensitivities. This challenge has become more prominent with the convergence of occupational skin health and environmental sustainability goals in healthcare settings worldwide, underscoring the need for alternative hand hygiene solutions that protect skin integrity whilst supporting compliance targets and staff well-being. Objectives: To assess healthcare workers' acceptance and tolerability of an organic bioethanol-based hand rub as a potential alternative to conventional alcohol-based hand sanitisers. **Methods:** A non-blinded study was conducted over one-month with 41 healthcare workers (29 nurses, 4 doctors, 6 ancillary staff, and 2 allied health workers) recruited through convenience sampling at Singapore General Hospital. Participants received a bioethanol-based hand rub (Saniswiss Sanitizer H1) containing bioethanol (72% concentration), moisturising agents, and super-fatting agents (perfume- and fragrance-free) in 100mL or 500mL bottles with ongoing replenishment. Data collection occurred at three time points: baseline (initial skin integrity assessment), 3-5 days post-implementation (follow-up questionnaire), and one month (final questionnaire and hand rub consumption calculation). Questionnaires were administered via FormSG platform with data analysed using Microsoft Excel. **Results:** Average consumption was 380mL per participant, with nurses demonstrating highest usage at 400mL. Initial skin assessment identified 17 participants with pre-existing dry skin and/or redness, which decreased to 6 participants reporting mild symptoms by study completion. User acceptance was high: 98% rated colour as pleasant or very pleasant, 95% approved of smell, 76% reported no sticky residue, 95% experienced no stinging sensation, 71% found the hand rub non-drying, and 90% rated drying speed as fast or very fast. Overall satisfaction reached 98% after 3-5 days and maintained 92% after one month. Notably, 82% believed the product could enhance hand hygiene compliance, and 68% preferred the bioethanol-based hand rub over conventional sanitiser. **Conclusion:** Bioethanol-based hand rub demonstrated excellent user acceptance and skin tolerability among healthcare workers. The significant reduction in skin-related complaints, combined with high satisfaction rates and improved skin condition despite frequent use, supports its potential implementation as an effective alternative hand hygiene solution for healthcare workers, particularly those with contact dermatitis. Figure 1: Progression of skin scale scores during study period Figure 2: Breakdown of Product Preferences Across Professional Group

